# Transcriptomic Profiling of Lesional and Perilesional Skin in Atopic Dermatitis Suggests Barrier Dysfunction, Inflammatory Activation, and Alterations to Vitamin D Metabolism

**DOI:** 10.3390/ijms26136152

**Published:** 2025-06-26

**Authors:** Teresa Grieco, Giovanni Paolino, Elisa Moliterni, Camilla Chello, Alvise Sernicola, Colin Gerard Egan, Mariangela Morelli, Fabrizio Nannipieri, Santina Battaglia, Marina Accoto, Erika Tirotta, Silvia Trasciatti, Silvano Bonaretti, Giovanni Pellacani, Stefano Calvieri

**Affiliations:** 1 Dermatology Clinic, Department of Clinical Internal, Anesthesiological and Cardiovascular Sciences, Sapienza University of Rome, 00185 Rome, Italy; teresa.grieco@uniroma1.it (T.G.); elisa.moliterni@gmail.com (E.M.); c.chello@unicampus.it (C.C.); alvise.sernicola@uniroma1.it (A.S.); giovanni.pellacani@uniroma1.it (G.P.); stefano.calvieri@uniroma1.it (S.C.); 2 Unit of Dermatology and Cosmetology, IRCCS Ospedale San Raffaele, 20132 Milan, Italy; 3 CE Medical Writing SRLS, 56021 Pisa, Italy; colingegan@gmail.com; 4 Fondazione Pisana per la Scienza, 56017 Pisa, Italy; mmorelli190977@gmail.com; 5 Clinical Research, Abiogen Pharma, 56121 Pisa, Italy; fabrizio.nannipieri@abiogen.it (F.N.); santina.battaglia@abiogen.it (S.B.); marina.accoto@abiogen.it (M.A.); erika.tirotta@abiogen.it (E.T.); 6 Galileo Research Srl, 56019 Pisa, Italy; silvia.trasciatti@galileoresearch.it (S.T.); silvano.bonaretti@galileoresearch.it (S.B.)

**Keywords:** atopic dermatitis, vitamin D receptor, skin barrier maintenance, gene expression

## Abstract

Atopic dermatitis (AD) is a chronic inflammatory skin disease marked by impaired barrier function and immune dysregulation. This study explores transcriptomic differences between lesional (IL) and perilesional (PL) skin in patients with AD, focusing on barrier-related and vitamin D-associated pathways. RNA sequencing was performed on matched IL and PL biopsies from 21 adults with moderate-to-severe AD. Differential gene expression, pathway enrichment, and correlation analysis with clinical variables were assessed. A total of 8817 genes were differentially expressed in IL versus PL skin (padj < 0.05). Among genes with the highest level of dysregulation, strong upregulation was observed for inflammatory mediators (*IL-19*, *IL-8*, *CXCL6*), and epidermal remodeling and barrier-disrupting genes (*MMP1*, *GJB2*). The vitamin D pathway genes *CYP27B1* and *CYP24A1* were also significantly upregulated. In contrast, key barrier-related genes such as *FLG2* and *CGNL1* were markedly downregulated. While some patterns in gene expression showed subgroup-specific trends, no independent clinical predictors emerged in multivariate models. Reactome pathway analysis revealed the enrichment of pathways involved in keratinization, cornified envelope formation, IL-4/IL-13 signaling, chemokine activity, and antimicrobial responses, highlighting coordinated structural and immunologic dysregulation in lesional skin. Lesional skin in AD displays a distinct transcriptomic profile marked by barrier impairment, heightened inflammatory signaling, and activation of vitamin D-related pathways. These findings provide the first RNA-seq-based comparison of IL and adjacent PL skin in AD. We identify subclinical activation in PL skin and vitamin D pathway upregulation with disrupted gene coordination in lesions. These findings enhance our understanding of the molecular mechanisms underlying inflammation in AD.

## 1. Introduction

Atopic dermatitis (AD) is a chronic inflammatory skin disease that affects both children and adults, characterized by relapsing eczematous lesions, pruritus, and a significant impact on quality of life [1]. The pathogenesis of AD involves a complex interplay of epidermal barrier dysfunction, immune dysregulation, microbial colonization, and genetic predisposition [2]. A hallmark of AD is the dominance of a type-2 immune response, mediated primarily by Th2 cells and associated cytokines such as interleukin (IL)-4, IL-13, and IL-31, which drive inflammation, IgE production, and pruritus [3,4]. In skin lesions, these immune pathways are markedly upregulated, with proteomic and transcriptomic data showing increased expression of Th2 cytokines (e.g., IL-13, CCL17), matrix metalloproteinases, and inflammatory mediators that promote epidermal hyperplasia and immune cell infiltration, including eosinophils [4,5,6]. Parallel to immune dysregulation, epidermal barrier impairment is a central feature of AD. The stratum corneum is often compromised due to the reduced expression of structural proteins such as filaggrin (FLG), filaggrin-2 (FLG2), loricrin [1], and components of the cornified envelope, as well as tight junction proteins such as CGNL1 and CLDN1 [7]. These abnormalities disrupt keratinocyte differentiation and compromise physical defense mechanisms, promoting transepidermal water loss and increased susceptibility to allergens and microbes [8]. The pathways involved in keratinization and cornified envelope formation are consistently found to be downregulated in lesional skin, and their expression is often inversely correlated with local Th2 cytokine activity [9].

In addition, vitamin D metabolism has emerged as a potentially important regulatory axis in AD. Active vitamin D (1,25-dihydroxyvitamin D) is produced locally in the skin through the action of the enzyme CYP27B1 and degraded by CYP24A1 [10]. This pathway plays a dual role: promoting keratinocyte differentiation and barrier repair, while also inducing antimicrobial peptides such as cathelicidin (LL-37), and modulating the immune response through the vitamin D receptor (VDR) [11,12]. A recent proteomic study by Grieco et al. (2024) [13] revealed that AD lesional skin displays higher expression of VDR, CYP27B1, CYP24A1, and CAMP compared to perilesional skin, particularly in patients with more severe disease, suggesting an attempt by the skin to counteract inflammation and microbial challenge.

A recent comprehensive review of Nakajima et al. [6] has emphasized that transcriptomic studies, particularly those using RNA-seq, have uncovered widespread dysregulation of immune pathways (Th2, Th17, Th22), epidermal differentiation, and barrier function in AD skin. Notably, both lesional and non-lesional skin show altered gene expression compared to healthy skin, but lesional samples exhibit additional activation of inflammatory cytokines and barrier-associated defects [6]. Multiple transcriptomic studies have analyzed lesional versus healthy skin [9,14] or non-lesional skin areas from the same patients in both pediatric and adult AD cohorts [15,16,17].

To date, very few studies have directly compared lesional (intralesional, IL) and perilesional (PL) skin from the same AD patients [13,18]. Grieco et al. [13] remains the only proteomic study to explicitly analyze IL vs. PL skin, demonstrating that lesional sites show increased expression of pro-inflammatory, tissue remodeling, and vitamin D-related proteins, including CYP27B1, CYP24A1, and vitamin D receptor (VDR), compared to adjacent uninvolved skin. Their findings suggest that PL skin exhibits an intermediate, yet molecularly distinct, profile reflective of localized subclinical activation. However, no transcriptomic study to date has comprehensively characterized the IL–PL axis in adult AD using paired RNA-sequencing.

In this study, we aimed to fill this gap by performing bulk RNA-seq on matched IL and PL skin biopsies from adult patients with moderate-to-severe AD. Our goal was to identify gene expression differences and coordinated pathway alterations across key biological domains, including inflammation, epidermal barrier integrity, and vitamin D metabolism, thus providing a transcriptome-level view of the spatial organization of disease activity within affected skin.

## 2. Results

### 2.1. Patient Characteristics

The clinical and demographic characteristics of the study population (*n* = 21) are summarized in Table 1. The majority of participants were male (61.9%), under 60 years of age (81.0%), with a mean BMI of 24.1 ± 4.1. All patients presented with moderate-to-severe atopic dermatitis (EASI ≥ 16). The most common clinical phenotype involved generalized disease (47.6%), followed by head/neck (33.3%) and flexural sites (14.3%).

Disease onset occurred during childhood in 71.4% of cases. Comorbid asthma was reported in 71.4% of participants, while 33.3% had a history of allergic rhino-conjunctivitis. Skin prick test positivity was observed in 38.1% of patients. Elevated total serum IgE levels (≥100 IU/mL) were found in 66.7% of subjects. Regarding vitamin D status, 60.0% had 25(OH)D levels below 30 ng/mL. No statistically significant associations were observed between vitamin D deficiency status (<20 vs. ≥20 ng/mL or <30 vs. ≥30 ng/mL, depending on the cutoff applied) and a range of clinical variables. 

### 2.2. Differential Gene Expression in Lesional and Perilesional Skin Biopses

**Figure 1:**
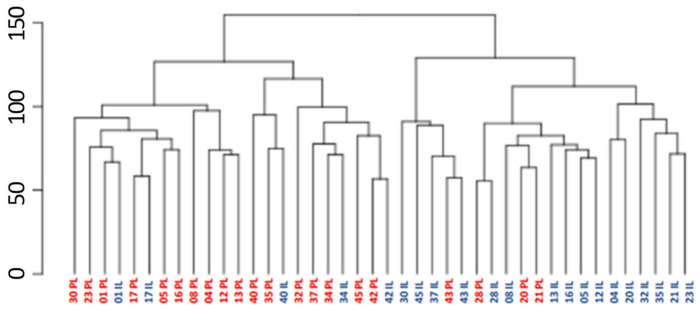
Hierarchical clustering of biopsy samples according to their similarity of expression data on all genes that received at least two reads. Expression data were rlog-transformed and the Euclidean distance was calculated. The number indicates the patient’s code. PL, Peri-lesional samples (denoted by red text); IL, Intra-lesional samples (denoted by blue text).

**Figure 2:**
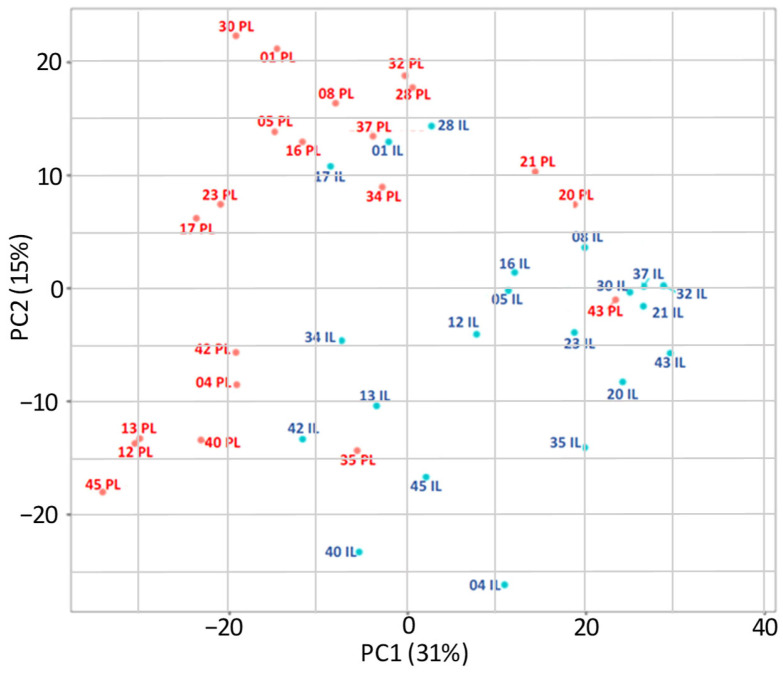
Principal component analyses of the rlog-transformed expression data of all genes that received at least two reads. The percentage values on the X- and Y-axes describe how much of the variance between samples is captured in this principal component. Samples are colored according to group. PL, Peri-lesional samples (denoted by red text); IL, Intra-lesional samples (denoted by blue text); PC = Principal component.

RNA-sequencing analysis revealed significant differences in gene expression between PL and IL skin biopsies, as illustrated by hierarchical clustering and principal component analysis (PCA) (Figure 1 and Figure 2 and Supplementary Figure S1).

**Figure 3:**
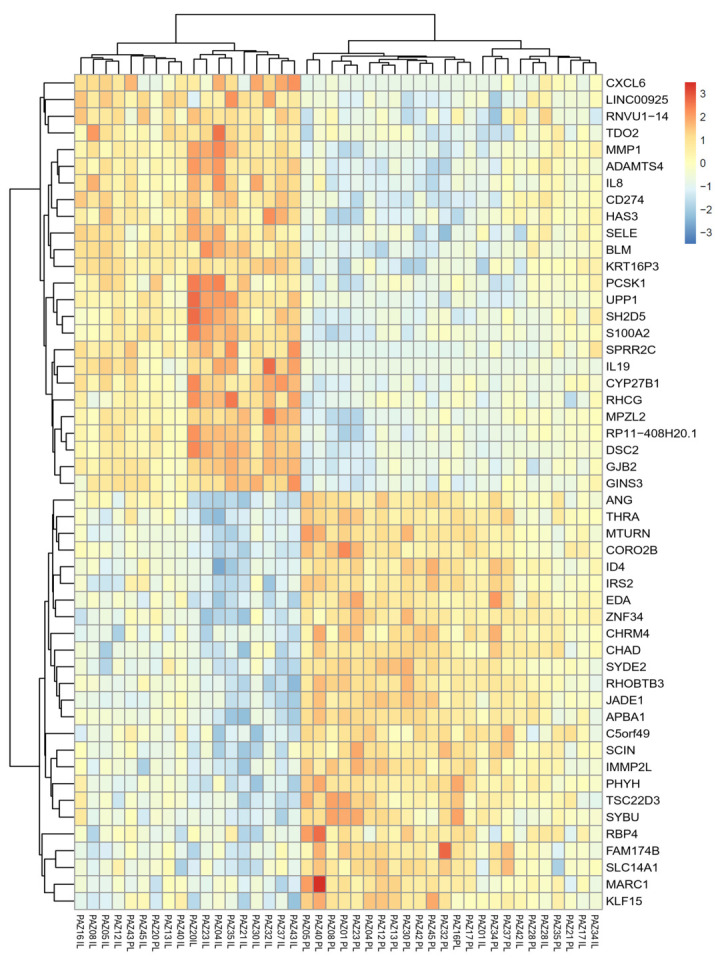
Color-coded heatmap representing the 50 most differentially expressed genes (25 upregulated, 25 downregulated) in intralesional (IL) versus perilesional (PL) skin biopsies from patients with AD, based on the following criteria: adjusted *p*-value (padj) < 0.05 and absolute log2FoldChange > 0.5. The z-score of normalized counts was used. The color scale represents the gene expression levels relative to the mean, with red indicating up-regulation (+3 z-score), blue indicating down-regulation (–3 z-score), and white representing no change (z = 0).

A total of 8817 genes were identified as significantly differentially expressed in IL samples compared to PL samples, based on an adjusted *p*-value (padj) < 0.05 (Supplementary Table S1). The log_2_ fold change values ranged from upregulation of +6.43 to downregulation of −4.23. Using more stringent criteria (padj < 0.05 and an absolute log_2_ fold change > 0.5), 50 of the most differentially expressed genes (DEGs) were selected, comprising the 25 most upregulated (highest positive log_2_FC) and the 25 most downregulated (lowest negative log_2_FC) transcripts in IL compared to PL samples (Figure 3, Supplementary Table S1). Notably, among these, a number of cytokines and chemokines, such as *IL-19* (log_2_FC = 6.43, *p* = 7.34 × 10^−14^), *IL8* (log_2_FC = 4.31, *p* = 6.04 × 10^−11^), and *CXCL6* (log_2_FC = 5.33, *p* = 6.51 × 10^−12^), were strongly upregulated in IL samples, reflecting an activated inflammatory environment. Likewise, *MMP1* (log_2_FC = 5.42, *p* = 1.41 × 10^−16^), a matrix metalloproteinase involved in tissue remodeling, and *GJB2* (log_2_FC = 2.72, *p* = 4.21 × 10^−13^), encoding connexin 26, a gap junction protein implicated in barrier regulation, showed elevated expression levels.

### 2.3. Expression Changes in Atopic Dermatitis-Related Genes

To further investigate the mechanisms underlying the observed gene expression changes, we focused on a subset of DEGs (Table 2), selected based on our previously published proteomic study in AD, which identified differentially expressed proteins related to skin barrier function and vitamin D metabolism in IL versus PL skin [13]. The involvement of these genes in epithelial barrier integrity, vitamin D metabolism, and immune response/inflammation has been previously reported elsewhere [19,20,21,22]. In total, eight genes were significantly differentially expressed (padj < 0.05). The most markedly downregulated genes were *FLG2* and *CGNL1*, whose expression was reduced by approximately 2.3-fold (log_2_FC = −1.17, *p* < 0.0001) and 2-fold (log_2_FC = −1.02, *p* < 0.0001), respectively (Table 2, Supplementary Figure S2), suggesting compromised skin integrity in lesional areas. In contrast, *CYP27B1* and *CYP24A1*, key enzymes involved in vitamin D metabolism, were upregulated by approximately 3.7-fold (log_2_FC = 1.89, *p* < 0.0001) and 3.5-fold (log_2_FC = 1.79, *p* < 0.0001), respectively (Table 2, Supplementary Figure S3), indicating a robust activation of the vitamin D pathway. Moreover, the antimicrobial peptide *CAMP*, involved in innate immune defense, showed a strong 5-fold upregulation (log_2_FC = 2.32, *p* = 0.0002) (Table 2, Supplementary Figure S2), consistent with enhanced immune activation in lesional skin. Other significantly but less markedly relevant DEGs included *CDH1*, *TJP1*, and *CTNNA1*, which were modestly upregulated by approximately 1.4-fold (log_2_FC = 0.48, *p* = 0.0001), 1.16-fold (log_2_FC = 0.21, *p* = 0.0083), and 1.13-fold (log_2_FC = 0.18, *p* = 0.016), respectively. In contrast, *CLDN1* showed a mild downregulation of approximately 1.36-fold (log_2_FC = −0.44, *p* = 0.0047). No significant differences were observed in the expression levels of *CTNNB1*, *OCLN*, and *VDR* (padj > 0.05) (Table 2, Supplementary Figure S2).

Co-expression analysis of genes involved in barrier function and vitamin D metabolism revealed fewer and weaker correlations in IL skin compared to PL skin (Supplementary Figure S3), possibly due to high interindividual variability or shifts in basal expression levels. Notably, in PL skin, *CYP27B1* showed strong positive correlations with several epithelial barrier genes, including *CTNNA1* (r = 0.76, *p* < 0.001), *CDH1* (r = 0.82, *p* < 0.001), and *TJP1* (r = 0.81, *p* < 0.001). In contrast, these associations were markedly attenuated in IL skin, where the correlations between *CYP27B1* and the same genes were weaker or only partially significant: *CTNNA1* (r = 0.76, *p* < 0.001), *CDH1* (r = 0.30, *p* = 0.181), and *TJP1* (r = 0.59, *p* = 0.005).

### 2.4. Association Between Gene Expression and Clinical Features of AD Patients

**Figure 4:**
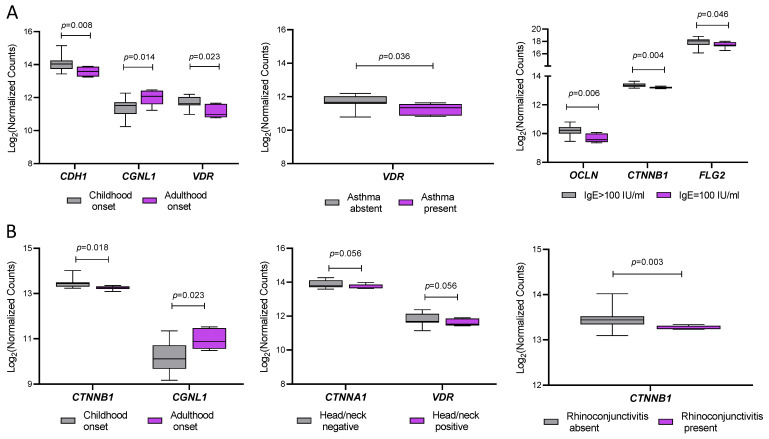
Sub-analysis of AD-related gene expression in relation to clinical features in perilesional, PL (**A**) and intralesional, IL (**B**) skin samples. (**A**) Expression levels of selected genes in PL skin stratified by disease onset (childhood vs. adulthood), comorbid asthma (present vs. absent), and total serum IgE levels (≤100 IU/mL vs. >100 IU/mL). (**B**) Gene expression in IL skin stratified by disease onset, head/neck lesion involvement (present vs. absent), and history of rhinoconjunctivitis. Gene expression is shown as log_2_ normalized counts. *p*-values indicate statistically significant differences between groups. Data are presented as box-and-whisker plots: boxes represent the interquartile range (IQR), horizontal lines indicate the median, and whiskers denote the minimum and maximum values).

To explore whether clinical features of AD patients were associated with the expression of specific genes in IL and PL skin, we performed subgroup analyses stratifying samples by relevant variables. In PL samples (Figure 4A), the expression of *CDH1* and *VDR* was significantly lower in patients with adult-onset disease compared to those with childhood-onset disease (*p* = 0.008 and 0.023, respectively), whereas *CGNL1* showed the opposite pattern (*p* = 0.014). Additionally, VDR expression was lower in patients with comorbid asthma (*p* = 0.036), and significantly reduced levels of *OCLN* (*p* = 0.006), *CTNNB1* (*p* = 0.004), and *FLG2* (*p* = 0.046) were observed in patients with total serum IgE ≥ 100 IU/mL.

In IL skin (Figure 4B), *CTNNB1* expression was significantly lower (*p* = 0.018) and *CGNL1* higher (*p* = 0.023) in patients with adult-onset disease. A trend toward lower expression of *CTNNA1* (*p* = 0.056) and *VDR* (*p* = 0.056) was observed in patients with head/neck involvement. Moreover, *CTNNB1* was significantly downregulated in patients with a history of rhinoconjunctivitis (*p* = 0.003). Despite these associations, univariate and multivariate linear regression analyses did not reveal any statistically significant relationships between gene expression and the clinical variables considered.

### 2.5. In Terminal Keratinocyte Differentiation and Epidermal Barrier Function (Table 3)

The “Keratinization” pathway, in particular, showed the upregulation of several key genes such as *KRT6A*, *KRT16*, and *SPRR2F*, all of which are strongly linked to epidermal stress responses and AD pathophysiology. A detailed list of differentially expressed genes within this pathway is provided in Table 4. In parallel, we observed significant enrichment of immune and inflammatory pathways, including “Interleukin-4 and Interleukin-13 signaling”, “Chemokine receptors bind chemokines”, and “Antimicrobial peptides” (Table 3).

**Table 3:** Most significantly enriched Reactome pathways by the observed gene expression changes.

FDR	*p*-Value	Pathway Name
6 × 10^−4^	1 × 10^−6^	Keratinization
6 × 10^−4^	3 × 10^−6^	Interleukin-4 and Interleukin-13 signaling
6.24 × 10^−3^	7.2 × 10^−5^	Antimicrobial peptides
9.23 × 10^−3^	1.04 × 10^−4^	Chemokine receptors bind chemokines
1.06 × 10^−2^	1.43 × 10^−4^	Formation of the cornified envelope
7.73 × 10^−2^	1.23 × 10^−3^	Calcitonin-like ligand receptors
8.62 × 10^−2^	1.57 × 10^−3^	Neutrophil degranulation
1.78 × 10^−1^	3.63 × 10^−3^	Regulation of TLR by endogenous ligand
2.36 × 10^−1^	5.90 × 10^−3^	NR1H2 and NR1H3 regulate gene expression linked to triglyceride lipolysis in adipose
2.36 × 10^−1^	5.90 × 10^−3^	Muscarinic acetylcholine receptors
2.78 × 10^−1^	7.51 × 10^−3^	Activation of matrix metalloproteinases
2.96 × 10^−1^	9.38 × 10^−3^	Collagen degradation
2.96 × 10^−1^	1.00 × 10^−2^	Interleukin-10 signaling
2.96 × 10^−1^	1.02 × 10^−2^	Defective SFTP2A causes IPF
3.26 × 10^−1^	1.21 × 10^−2^	TRKA activation by NGF
4.01 × 10^−1^	1.61 × 10^−2^	Beta defensins
4.01 × 10^−1^	1.67 × 10^−2^	Hormone ligand-binding receptors
4.47 × 10^−1^	2.03 × 10^−2^	Retinoid metabolism disease events
4.47 × 10^−1^	2.03 × 10^−2^	Defective SLC26A4 causes Pendred syndrome (PDS)
6.07 × 10^−1^	3.04 × 10^−2^	Defective SLC17A8 causes autosomal dominant deafness 25 (DFNA25)
6.07 × 10^−1^	3.04 × 10^−2^	Defective SLC5A5 causes thyroid dyshormonogenesis 1 (TDH1)
6.91 × 10^−1^	3.64 × 10^−2^	NR1H2 and NR1H3 regulate gene expression linked to gluconeogenesis
7.24 × 10^−1^	4.02 × 10^−2^	Defensins
7.73 × 10^−1^	4.88 × 10^−2^	Assembly of active LPL and LIPC lipase complexes

FDR, false discovery rate.

**Table 4:** Genes and proteins mapped to the keratinization pathway. Log2FoldChange indicated the upregulation of genes as positive values and the downregulation of genes as negative values, in biopsy IL vs. biopsy PL comparison.

Function	UniProt Id	padj	*p*-Value	Log2FoldChange	Gene Name	Gene_id
Stress-inducible keratin, structural role in epidermal repair	P04259	1.8 × 10^−6^	<1 × 10^−4^	4.357463	*KRT6C*	ENSG00000170465
Cornified envelope protein, reinforces barrier	Q9UBC9	1.63 × 10^−3^	1.42 × 10^−4^	3.469405	*SPRR3*	ENSG00000163209
Stress-inducible keratin, part of intermediate filaments	P04259	8 × 10^−7^	<1 × 10^−4^	3.323752	*KRT6B*	ENSG00000185479
Cornified envelope protein, cross-linker	Q96RM1	1.01 × 10^−3^	7.26 × 10^−5^	3.158956	*SPRR2F*	ENSG00000244094
Stress-inducible keratin, part of intermediate filaments	P08779	9.49 × 10^−5^	2.6 × 10^−6^	3.117222	*KRT16*	ENSG00000186832
Protease inhibitor (elafin), limits inflammation/desquamation	P19957	5.93 × 10^−5^	1.3 × 10^−6^	3.056438	*PI3*	ENSG00000124102
Stress-inducible keratin, part of intermediate filaments	P02538	4.46 × 10^−5^	9 × 10^−7^	2.984315	*KRT6A*	ENSG00000205420
Serine protease inhibitor, regulates corneodesmosome degradation	Q6UWN8	4.16 × 10^−3^	5.09 × 10^−4^	2.808229	*SPINK6*	ENSG00000178172
Keratins in differentiating non-cornified epithelium	P12035	8.39 × 10^−5^	2.1 × 10^−6^	2.564823	*KRT3*	ENSG00000186442
Stress-inducible keratin, part of intermediate filaments	Q04695	1.6 × 10^−6^	<1 × 10^−4^	2.393522	*KRT17*	ENSG00000128422
Keratin-associated protein, stabilizes keratin filaments	P60370	4.67 × 10^−2^	1.3 × 10^−2^	−2.199704	*KRTAP10-5*	ENSG00000241123
Keratin-associated protein, stabilizes keratin filaments	Q6L8G5	2.66 × 10^−3^	2.72 × 10^−4^	−2.257585	*KRTAP5-10*	ENSG00000204572
Keratin-associated protein, stabilizes keratin filaments	Q6L8H1	1.7 × 10^−2^	3.38 × 10^−3^	−2.299814	*KRTAP5-4*	ENSG00000241598
Keratin-associated protein, stabilizes keratin filaments	P60014	3.08 × 10^−2^	7.48 × 10^−3^	−2.405668	*KRTAP10-10*	ENSG00000221859
Keratin-associated protein, stabilizes keratin filaments	Q9BYR3	1.87 × 10^−2^	3.84 × 10^−3^	−2.601125	*KRTAP4-4*	ENSG00000171396

Gene_id: Ensembl Gene Identifier; padj: Adjusted *p*-value using the Benjamini–Hochberg method to correct for multiple testing (False Discovery Rate); UniProt Id: Unique protein identifier from the UniProt database.

## 3. Discussion

In this study, transcriptomic profiling of IL and PL skin samples from adult AD patients revealed extensive molecular alterations in lesional tissue. IL skin showed significant upregulation of inflammatory cytokines, chemokines, matrix remodeling enzymes, and vitamin D metabolism-related genes, alongside the downregulation of key epidermal barrier components. Co-expression network analysis indicated a loss of transcriptional coordination in IL samples, particularly among barrier- and vitamin D-related genes. Pathway enrichment confirmed the simultaneous activation of immune signaling and the suppression of structural epidermal programs, highlighting the integrated immune-barrier dysregulation that defines AD lesions.

To our knowledge, this is the first RNA-seq-based study to directly compare IL and anatomically adjacent PL skin in adult AD. This design was selected to capture spatially localized transcriptional changes that may be masked in broader comparisons, such as IL versus distant non-lesional or healthy skin. Clinically normal-appearing skin in AD is known to hold molecular abnormalities [23], and PL skin represents a biologically relevant zone of early or subclinical activation within the lesional microenvironment. Prior studies, including Grieco et al. (2024) [13], have shown that PL skin differs from both IL and distant uninvolved sites in protein expression, particularly in inflammatory, remodeling, and vitamin D-related pathways, suggesting that PL skin is not simply a passive control but reflects early pathophysiological changes. Physiological measurements (e.g., altered hydration, pH) in PL skin further support its distinct status [24]. Using paired biopsies from the same individual allowed us to control for inter-patient variability in genetic background, systemic immune homeostasis, and environmental exposure. This within-subject design increases our sensitivity to detect true disease-related transcriptomic changes, an advantage that is often lost in between-subject comparisons involving distant, uninvolved, or healthy skin. Moreover, while non-lesional skin has been studied previously [6,25], direct IL–PL transcriptomic comparisons remain scarce. In this regard, the present findings offer novel insights into the spatial architecture of AD inflammation and help define PL skin as a transitional, molecularly active zone that bridges healthy and overtly inflamed tissue.

Transcriptomic profiling of IL and PL skin samples from adult AD patients revealed marked transcriptional differences, as reflected by the clear clustering of IL and PL samples. In total, 8817 genes were identified as significantly differentially expressed between IL and PL samples using a padj < 0.05. By comparison, Dessie et al. (2024) [26] reported 441 DEGs in lesional vs. non-lesional AD skin. This discrepancy likely reflects methodological differences, as their study focused on co-expression networks and applied stricter inclusion criteria based on correlation strength (|R| ≥ 0.5) in addition to nominal *p*-values.

Despite these differences, both studies converge on the conclusion that lesional AD skin exhibits extensive molecular remodeling relative to non-lesional tissue. In addition, the high DEG count in our study is biologically plausible given AD’s complex pathophysiology [23,27,28]. However, the authors acknowledge that DEG numbers are highly dependent on the stringency of selection criteria; applying a moderate fold-change threshold (|log_2_FC| > 0.5) significantly reduced the DEG count. Importantly, all RNA samples were processed in a single experimental batch, and PCA analyses showed no evidence of batch effects, supporting the validity of the findings.

Tsoi et al. [14] further supported this concept, demonstrating that non-lesional AD skin is not molecularly normal but displays mild pro-inflammatory and epidermal changes that are greatly intensified in lesions. Moreover, Möbus et al. [9] described a robust “core” eczema signature shared by both lesional and non-lesional skin, comprising the altered expression of genes involved in keratinocyte differentiation and immune activation, with additional enrichment of inflammatory pathways in lesional areas.

Together, these studies reinforce the concept that AD lesions are defined by a unique and amplified disease-specific molecular program, distinct from the surrounding clinically uninvolved skin. Although they have primarily compared lesional to non-lesional skin, our direct comparison of IL and PL samples provides novel insight into the molecular heterogeneity within affected areas. By focusing on PL skin, a clinically uninvolved but spatially adjacent compartment, our study captures a more detailed view of the transcriptomic transition from subclinical to overt inflammation

Recent human omics studies have clearly demonstrated that AD lesions exhibit marked overexpression of pro-inflammatory cytokines, chemokines, and tissue remodeling enzymes [6,15,29]. In our dataset, lesional skin showed strong upregulation of cytokine genes such as *IL-19* and *IL-24*, chemokines like *CXCL6*, and matrix metalloproteinases, including *MMP1*. The elevated expression of cytokines such as *IL-4*, *IL-13*, *IL-19*, and *IL-22* has been widely reported in AD skin and correlates with disease severity and inflammation [30]. These cytokines contribute to the characteristic features of AD, including eosinophilic infiltration, epidermal hyperplasia, pruritus, and microbial dysbiosis. IL-24, in particular, is produced by Th2 cells and keratinocytes in response to type 2 cytokines and plays a role in promoting skin barrier dysfunction, hyperplasia, inflammation, itch, and colonization by Staphylococcus aureus [31]. The concurrent overexpression of MMPs, especially MMP1, reflects active tissue remodeling. Zhu et al. [32] identified *MMP1* as one of the most consistently upregulated genes in a meta-analysis of seven AD gene expression datasets comprising lesional and non-lesional biopsies. Recent findings by Wang et al. [33] demonstrated that keratinocytes exposed to Staphylococcus aureus, a bacterium frequently colonizing AD skin, respond by upregulating *MMPs*, thereby linking microbial colonization to enhanced proteolytic and inflammatory responses. Together, these data and the recent literature reinforce the view of AD lesions as zones of pronounced immune activation and protease-driven matrix remodeling, perpetuating the chronic inflammatory cycle.

In parallel with the inflammatory response, we observed significant downregulation of barrier-related genes in IL samples, including *FLG2* and *CGNL1*. Impaired expression of these cornified envelope components is a well-established hallmark of AD pathology [34]. We also noted reduced transcripts of *CLDN1*, a tight junction protein essential for maintaining epidermal integrity. *CLDN1* is known to be diminished in both lesional and non-lesional AD skin, with expression inversely correlated with local inflammation [19]. Chronic Th2-driven inflammation plays a direct role in this barrier defect, as IL-4, IL-13, and IL-31 suppress keratinocyte differentiation and junctional gene expression [35]. Specifically, IL-4/IL-13 signaling downregulates FLG2, loricrin, involucrin, and CLDN1, thereby disrupting both the stratum corneum and tight junctions [35]. The cumulative effect is a weakened skin barrier in lesional tissue, which increases transepidermal water loss and facilitates the penetration of environmental allergens and microbial agents [36]. This barrier defect, in turn, promotes immune activation, creating a self-reinforcing inflammatory loop. The “outside-in and inside-out” paradigm has been widely recognized as central to AD pathogenesis, where barrier defects initiate immune responses, and ongoing inflammation exacerbates epidermal dysfunction [35]. The reduced expression of barrier-associated genes in IL skin that we observed provides molecular confirmation of this vicious cycle and is consistent with the clinical manifestations of xerosis, increased sensitivity, and barrier impairment in AD patients.

An important finding from our analysis was the significant upregulation of the vitamin D metabolism genes *CYP27B1* and *CYP24A1* in AD lesions. The simultaneous increase in both enzymes suggests enhanced local vitamin D metabolic activity in lesional skin. This observation is supported by a recent proteomic study, which found higher expression levels of CYP27B1, CYP24A1, and the VDR in IL skin of AD patients compared to PL areas [13]. Notably, higher EASI scores were associated with the increased expression of these enzymes and the antimicrobial peptide CAMP, suggesting a compensatory activation of the vitamin D pathway during severe inflammation [13]. Our transcriptomic results corroborate these observations at the mRNA level, supporting the hypothesis that lesional skin may activate the vitamin D pathway as an endogenous mechanism to enhance antimicrobial defense and support barrier restoration. In response to injury or infection, keratinocytes upregulate *CYP27B1* and *VDR*, leading to increased local production of active vitamin D, which in turn induces *CAMP* expression, thereby strengthening cutaneous immunity [37]. Clinically, vitamin D sufficiency has been linked to improved AD outcomes, and supplementation studies have shown increased lesional *CAMP* expression and reduced disease severity [38]. These results provide molecular evidence for the beneficial role of vitamin D in AD. However, the simultaneous increase in CYP24A1, which degrades active vitamin D, may limit this protective effect, suggesting a complex regulatory balance. The functional consequences and regulatory mechanisms behind this upregulation remain to be fully elucidated and warrant further investigation.

In adult-onset AD, we observed upregulation of the tight junction gene *CGNL1* and downregulation of the adherens junction genes *CDH1* (in PL) and *CTNNB1* (in IL), consistent with chronic epithelial remodeling [3,18,39]. *VDR* was selectively reduced in perilesional skin, particularly in patients with asthma or head/neck involvement, suggesting early impairment of vitamin D signaling [13,40]. Allergic comorbidities such as high IgE and rhinoconjunctivitis were associated with decreased *CTNNB1*, *OCLN*, and *FLG2* in PL skin, indicating subclinical barrier weakness that may facilitate sensitization [19,41,42]. These findings support a model where non-lesional dysregulation of barrier genes contributes to systemic atopy, while inflammation in lesional skin may conceal these alterations.

Co-expression analysis revealed coordinated expression of vitamin D metabolism and barrier-related genes in perilesional skin, with *CYP27B1* showing strong positive correlations with *CDH1*, *CTNNA1*, and *TJP1*. In lesional skin, these relationships were diminished, suggesting inflammation-driven disruption of gene network organization, consistent with prior studies showing altered co-expression architecture in AD lesions [43].

Compared to the findings of Grieco et al. [13], which analyzed the same atopic dermatitis–associated targets from a proteomic perspective using antibody microarrays, our RNA-seq-based transcriptomic sub-analysis revealed both overlapping and divergent molecular signatures across clinical subgroups. Discrepancies between gene and protein expression are a well-recognized phenomenon in molecular biology, particularly in complex diseases such as AD [44]. For example, Wilhelm et al. [45] reported a correlation coefficient of approximately 0.4 between mRNA and protein levels across human tissues, indicating that transcript abundance explains only ~16% of protein-level variance. Similarly, Perl et al. [46] showed that protein expression is often more conserved than mRNA levels across tissues, suggesting that post-transcriptional regulation plays a major role. This limited correlation is especially pronounced in genes involved in immune signaling, epithelial homeostasis, and barrier function, where protein levels are shaped by additional layers of regulation such as mRNA stability, translational efficiency, and protein turnover [44,47].

Consistently, proteomic studies in AD have identified elevated levels of keratins, S100 proteins, and metabolic enzymes that are not always mirrored at the transcript level—as seen in the differences between Goleva et al. [48] and transcriptomic studies such as Cole et al. [49]. Recent reviews [50,51] reinforce this notion, highlighting that significant changes in mRNA abundance do not necessarily result in proportional shifts in protein expression, and vice versa. These findings emphasize the importance of integrating proteomic and transcriptomic data to fully capture the complexity of molecular dysregulation in AD and avoid the limitations of single-layer analyses.

Another key distinction lies in patient populations. All individuals in our cohort had moderate-to-severe AD (EASI ≥16), representing a more homogeneous, high-severity subgroup. In contrast, Grieco et al. included a broader severity spectrum. This difference likely contributed to the more marked gene expression stratification observed in our dataset—particularly in PL skin—whereas in Grieco, some barrier-related proteins appeared upregulated in lesional areas, possibly reflecting chronic tissue adaptation. Finally, while both studies highlighted the relevance of junctional and vitamin D-related pathways, our RNA-based approach allowed the detection of subtle, early transcriptional shifts (e.g., VDR downregulation in PL skin with asthma or head/neck involvement), which may precede protein-level compensation.

Together, these findings highlight the complementarity of proteomic and transcriptomic approaches in dissecting AD pathogenesis and support the use of multi-omics profiling to better define clinical–molecular endotypes.

Pathway analysis of the 8817 DEGs in IL skin revealed two major categories of dysregulation: epidermal structural processes and immune/inflammatory responses. Pathways enriched in IL samples included those related to keratinization and cornified envelope formation, reflecting abnormalities in keratinocyte differentiation and epidermal barrier assembly. Concurrently, we observed the significant enrichment of pathways associated with IL-4/IL-13 signaling, chemokine activity, and antimicrobial peptide expression, indicating active immune involvement. This perturbation of barrier and immune pathways is a hallmark of AD and has been reported in other transcriptomic studies [6,52]. This dual signature supports the well-established concept that AD pathogenesis is driven by both “outside-in” (barrier-driven) and “inside-out” (immune-driven) mechanisms, acting in concert to compromise the epithelial barrier [53].

The recent literature further supports the interdependence between epidermal barrier dysfunction and Th2-driven inflammation in AD [30]. On the one hand, inherited or acquired barrier defects—such as filaggrin deficiency, impaired cornification, or tight junction disruption—render the skin more susceptible to immunologic sensitization. On the other hand, chronic Th2/Th22-mediated inflammation suppresses the expression of key structural proteins (e.g., filaggrin, claudins) and disrupts lipid metabolism, further weakening the barrier [52]. This self-perpetuating loop of inflammation and structural impairment contributes to disease chronicity and flare-ups. Altogether, our findings support a model in which immune activation and barrier abnormalities are not independent events but represent tightly interconnected pathological processes in AD lesions. Moreover, consistent with our findings, Antonatos et al. [43] have recently reinforced the molecular distinctiveness of lesional skin by combining transcriptome-wide association studies, differential expression meta-analyses, and co-expression networks. Their results identified the key regulator genes involved in both inflammatory signaling and keratinization (e.g., *LCE3E*, *LCE3D*) specifically in lesional skin.

Together, these findings describe a lesional transcriptional landscape marked by the breakdown of the skin barrier, activation of inflammatory and antimicrobial pathways, and upregulation of vitamin D metabolism genes. The disruption of gene co-regulation in lesional skin further highlights the complexity of AD pathophysiology and highlights potential molecular targets for future therapeutic strategies.

### 3.1. Study Limitations

The limitations of this study are first the sample size; although adequate for paired transcriptomic comparisons, it limits the statistical power to detect associations with clinical subgroups or disease severity. Although sex-based differences in gene expression have been described in other contexts, neither univariate nor multivariate analyses revealed any significant sex-associated effects in our dataset. Nevertheless, the unbalanced sex distribution remains a limitation and should be addressed in future studies with larger, sex-stratified cohorts. Second, RNA sequencing was performed on bulk tissue, which precludes cell-type-specific resolution and may mask contributions from minor but functionally relevant cell populations. Future single-cell or spatial transcriptomic approaches could provide more granular insights into the cellular origin of the observed transcriptional changes. Third, the analysis was restricted to adult patients with moderate-to-severe AD; thus, the findings may not fully generalize to milder disease phenotypes. Fourth, subgroup analyses based on clinical features (e.g., age of onset, IgE levels, asthma) were exploratory in nature and underpowered due to the small sample size. While they offer preliminary insights, these hypothesis-generating findings require validation in larger, independent cohorts. Fifth, a key limitation of the analysis is that, despite the paired biopsy design, statistical pairing (e.g., including patient ID as a blocking factor) was not applied in the differential expression model. While this choice simplified downstream analysis and was supported by robust IL vs. PL separation in PCA, it may have limited our ability to fully control for inter-individual variability. Last, although vitamin D pathway activation was observed at the transcriptomic level, protein validation and functional assays are needed to confirm biological activity and define its clinical relevance.

### 3.2. Conclusions

In this exploratory study, we conducted a transcriptome-wide analysis of IL and PL skin in adults with moderate-to-severe AD. This within-patient design captured local disease-related transcriptional changes while reducing inter-individual variability. To our knowledge, this is the first RNA-seq study directly comparing IL and PL skin in AD.

This transcriptomic analysis highlights key molecular differences between IL and PL skin in adult atopic dermatitis. IL skin showed increased expression of inflammatory and vitamin D-related genes, along with reduced expression of genes involved in skin barrier function.

We identified three novel findings: (1) PL skin showed an intermediate transcriptomic profile, indicating subclinical activation and spatial disease extension beyond visible lesions; (2) IL skin showed significant upregulation of vitamin D metabolism genes (CYP27B1, CYP24A1, CAMP), suggesting a local inflammatory response; and (3) co-expression between CYP27B1 and key barrier genes was disrupted in IL skin, reflecting breakdown of coordinated gene regulation.

These findings reflect the combined effects of immune activation and structural disruption that define AD lesions. The comparison of matched skin samples also reveals early changes in nearby, unaffected areas, suggesting a gradient of disease activity. While the upregulation of antimicrobial and vitamin D pathway genes may represent a local response to inflammation, further studies are needed to clarify their functional role.

Overall, this study provides new insights into the localized molecular changes in AD and supports the development of targeted treatments that address both inflammation and barrier repair.

## 4. Materials and Methods

### 4.1. Sample Collection and Ethics Approval

Skin biopsies were collected from patients enrolled at Azienda Ospedaliera Universitaria Policlinico Umberto I, UOS Dermatologia Allergologica Professionale e Malattie Sessualmente Trasmesse. The study protocol was approved by the relevant ethical review board, and all participants provided written informed consent. A total of 42 skin biopsy samples were obtained from both IL and PL sites of patients diagnosed with atopic dermatitis. The inclusion and exclusion criteria, as well as the main patient characteristics, have been detailed elsewhere [18]. In summary, the study enrolled male and female participants aged 18 years or older diagnosed with moderate–severe atopic dermatitis.

The exclusion criteria encompassed other inflammatory or autoimmune skin conditions, disorders affecting calcium or bone metabolism, recent use of corticosteroids, serious systemic illnesses, specific infections, history of organ transplantation, cognitive impairments (e.g., dementia, psychosis), substance abuse, pregnancy, anticoagulant therapy, and recent exposure to sunlight. Participants were not subjected to dietary limitations.

During the initial screening, demographic data (sex, age, BMI), clinical history, details on AD onset and phenotype, disease severity (evaluated using the Eczema Area and Severity Index—EASI [54]), and presence of comorbid allergic conditions such as asthma or allergic rhinitis were recorded. Blood samples were also obtained at this visit. Within ten days, participants returned for additional assessments, including skin biopsy procedures for the analysis of protein expression. The study received authorization from the Director of the Dermatological Clinic, Policlinico Umberto I, Sapienza University of Rome, and was conducted in compliance with the Declaration of Helsinki, Good Clinical Practice (GCP) standards, International Council for Harmonisation (ICH) guidelines, and national regulations. Ethical approval was granted by the institutional ethics committee (protocol ID: DERM/AT/01).

### 4.2. Biopsy Sampling

During the initial evaluation, each patient with AD underwent two skin biopsies: one taken directly from the lesion (intralesional, IL) and another from the peri-lesional area, approximately 3 cm away from the lesion edge, as previously reported [18]. Following antiseptic cleansing with povidone iodine, local anesthesia was achieved using 2% mepivacaine. A tissue specimen up to 10 × 5 mm in size was excised. The wound was then sutured using 1–3 nylon (3.0) stitches and covered with a medicated dressing. Patients received prophylactic antibiotics (amoxicillin 1 g with clavulanic acid) every 12 h for five days. Suture removal was scheduled between 7 and 15 days after the procedure.

### 4.3. RNA Extraction and Quality Control

The total RNA was extracted from skin biopsy samples using the TRIzol reagent (Invitrogen) according to the manufacturer’s protocol, followed by purification with the RNeasy Mini Kit (Qiagen) to ensure high RNA integrity. RNA library preparation was performed using the KAPA RNA Hyperprep Kit with RiboErase (HMR) (Roche), following the manufacturer’s instructions. The quantity of RNA used for library preparation was 100 ng.

The quality and concentration of RNA were assessed using a Qubit RNA assay and a Bioanalyzer RNA assay. The RNA integrity number (RIN) and concentrations were evaluated to determine if they met the requirements for downstream analysis. All samples were sequenced using an Illumina NovaSeq 6000 platform with a paired-end read length of 2 × 100 bp. The sequencing parameters were established at a minimum Q30 value > 91.73%. All RNA samples were extracted, prepared, and sequenced as part of a single experimental batch using standardized protocols and the same Illumina NovaSeq 6000 platform.

### 4.4. RNA Sequencing and Data Processing

Raw sequencing data were generated as FASTQ files, consisting of paired-end reads. Adapter sequences were removed, and quality trimming was performed using FastQC (version 0.11.5-cegat), then the read data were trimmed accordingly. The reads were then aligned to the reference genome using a bioinformatics pipeline. The aligned reads are provided in BAM format, with corresponding index files (BAI).

### 4.5. Differential Gene Expression Analysis

Differential expression analysis (using a negative binomial generalized linear model to test for differential expression based on gene counts) between groups was performed using DESeq2 (version 1.24.0) in R (version 3.6.1) (R Core Team 2015). Raw counts derived from the mapping contained the number of reads mapped to each geneID.

Based on the read numbers, normalized counts were calculated. In the first normalization step, a fictive “reference sample” was calculated by DESeq2, defined as the geometric mean for each gene across all samples, regardless of group affiliation. Counts for each gene and sample were then divided by this reference value. In the second step, the size factor was estimated for each sample by calculating the median of these ratios. To obtain normalized counts, for each gene and sample, the raw counts were divided by the sample’s size factor. This normalization process accounted for different library sizes and for biases if, e.g., in some samples, only a few genes are highly expressed. Genes with less than two reads over all samples were omitted, improving the detection power and making multiple testing adjustments of *p*-values less stringent. Using normalized counts, the log2 fold change was calculated using DESeq2, where the *p*-value represents the statistical significance of this result (Wald test). Since we compared thousands of genes, multiple testing was accounted for using the Benjamini–Hochberg correction (implemented in DESeq2 to adjust the *p*-value; padj). The padj-value was used to determine significant differences in gene expression. Although IL and PL biopsies were biologically paired, differential expression analysis was performed using an unpaired model (~group) without including patient ID as a blocking factor, in order to maintain compatibility with downstream analyses and account for the study’s moderate sample size.

### 4.6. Data Visualization and Statistical Analysis

The clinical characteristics of the patients are presented as absolute numbers and percentages. Hierarchical clustering and principal component analysis (PCA) were employed to visualize relationships between samples based on gene expression data. For both analyses, variance-stabilizing transformation (rlog) was applied to the raw counts to reduce heteroscedasticity and ensure the equal contribution of all genes to the sample distances, particularly suited for datasets with small sample sizes. The PCA plot displays the two principal components that explain the highest proportion of variance between the samples.

FASTQ file quality was assessed using FastQC (version 0.11.5-cegat). All plots were generated in R (version 3.6.1) using the ggplot2 and dendextend packages.

Pathway enrichment analysis was performed using the Reactome Pathway Database (https://reactome.org), which provides curated information on biological pathways and reactions in human biology. The analysis was based on the list of differentially expressed genes (DEGs), including both up- and downregulated genes. Over-representation analysis was conducted using the hypergeometric test implemented in the Reactome online tool, which evaluates whether a given pathway contains more DEGs than expected by chance. *p*-values were adjusted for multiple testing using the Benjamini–Hochberg false discovery rate (FDR) correction. Only pathways with adjusted *p* < 0.05 were considered significantly enriched. Non-human identifiers were mapped to their human equivalents prior to analysis. As shown in Table 3, the analysis identified pathways relevant to epithelial barrier function, immune signaling, and vitamin D metabolism.

Statistical analyses were conducted using appropriate methods based on variable type and distribution. Fisher’s exact test was used to assess potential associations between vitamin D deficiency status (<20 vs. ≥20 ng/mL or <30 vs. ≥30 ng/mL, depending on the cutoff applied) and several binary categorical clinical variables, including sex (male vs. female), age group (<60 vs. ≥60 years), disease onset (childhood vs. adulthood), comorbidities (presence vs. absence of asthma and rhino-conjunctivitis), and sensitization status (positive vs. negative skin prick test, and total IgE < 100 vs. ≥100 IU/mL).

Comparisons between binary and continuous variables were performed using the Mann–Whitney U test. The relationships between continuous variables were evaluated using simple and multiple linear regression. Binomial logistic regression (both univariate and multivariate) was used to assess associations between continuous predictors and binary outcomes. Specifically, the Pearson correlation coefficient was used to assess potential univariate associations between the 12 genes involved in specific biological processes (epithelial barrier, vitamin D metabolism, and immune response/inflammation) (see Table 2) and continuous clinical variables (i.e. age, BMI, vitamin D levels); whereas, we used stepwise (forward) multivariate logistic regression analysis to evaluate the potential association between binary categorical clinical variables (see above) and the 12 genes. Separate models were run for each clinical variable. Spearman’s rank correlation coefficient was used to explore co-expression relationships between selected genes, accounting for non-normal distributions. All statistical tests were two-tailed, and statistical significance was defined as *p* < 0.05. Statistical analysis was performed using MedCalc Software (version 23.0.2, Broekstraat, 9030 Mariakerke, Belgium) or Graphpad Prism (version 9.0, GraphPad Software, Inc., San Diego, CA, USA).

## Figures and Tables

**Table 1:** Clinical characteristics of AD patients (*n* = 21).

Characteristic	*n* (%)
Gender, *n* (%)	
Male	13 (61.9)
Female	8 (38.9)
Age	
<60 years	17 (81.0)
≥60 years	4 (19.0)
BMI (Kg/m^2^), mean ± SD	24.1 ± 4.1
EASI score	
Mild (EASI < 16)	0 (0.0)
Moderate-to-severe (EASI ≥ 16)	21 (100)
Phenotype (localisation), *n* (%)	
Flexural sites	3 (14.3)
Generalised	10 (47.6)
Head/neck	7 (33.3)
Hands	1 (4.8)
^§^ Age of disease onset, *n* (%)	
Childhood	15 (71.4)
Adulthood	6 (28.6)
Asthma, *n* (%)	
Present	15 (71.4)
Absent	6 (28.6%)
Rhino conjunctivitis, *n* (%)	
Present	7 (33.3)
Absent	14 (66.7)
Skin prick test, *n* (%)	
Present	8 (38.1)
Absent	13 (61.9)
Total IgE (IU/mL), *n* (%)	
<100 IU/mL	7 (33.3)
≥100 IU/mL	14 (66.7)
* 25(OH)D vitamin D	
≥30 ng/mL	8 (40.0)
<30 ng/mL	12 (60.0)

EASI = Eczema Area and Severity Index. BMI, Body Mass Index. ^§^ Adult onset of AD was defined as diagnosis of AD at the age of 18 years. Data are presented as numbers and % or mean and SD. * one patient did not have 25(OH)D measured.

**Table 2:** Genes involved in specific biological processes (epithelial barrier, vitamin D metabolism, and immune response/inflammation) are differentially expressed intralesionaly with respect to perilesional biopsies.

Gene Name	Significance	padj	*p*-Value	Log2 Fold Change	Gene	Ensemble Gene id	
E-Caderin 1	S	1.30 × 10^−3^	1 × 10^−4^	0.4809	*CDH1*	ENSG0000039068	Epithelial Barrier
Zonulin 1	S	3.32 × 10^−2^	8.30 × 10^−3^	0.2136	*TJP1*	ENSG00000104067	
Alpha 1_Catenin	S	4.28 × 10^−2^	1.16 × 10^−2^	0.1762	*CTNNA1*	ENSG00000044115	
Beta 1_Catenin	NS	2.88 × 10^−1^	1.54 × 10^−1^	0.0797	*CTNNB1*	ENSG00000168036	
Claudin	S	2.17 × 10^−2^	4.70 × 10^−3^	−0.4409	*CLDN1*	ENSG00000163347	
Cingulin like	S	<1 × 10^−4^	<1 × 10^−4^	−1.0219	*CGNL1*	ENSG00000128849	
Filaggrin 2	S	2 × 10^−4^	<1 × 10^−4^	−1.1688	*FLG2*	ENSG00000143520	
Occludin	NS	6.5 × 10^−1^	5 × 10^−1^	−0.0853	*OCLN*	ENSG00000197822	
Cytochrome P450 Family 27 Subfamily B Member 1	S	<1 × 10^−4^	<1 × 10^−4^	1.8934	*CYP27B1*	ENSG00000111012	Vitamin D metabolism
Cytochrome P450 Family 24 Subfamily A Member 1	S	3 × 10^−4^	<1 × 10^−4^	1.7887	*CYP24A1*	ENSG0000019186	
Vitamin D Receptor	NS	2.37 × 10^−1^	1.17 × 10^−1^	0.1863	*VDR*	ENSG00000111424	
Cathelicidin Antimicrobial Peptide	S	2 × 10^−3^	2 × 10^−4^	2.3195	*CAMP*	ENSG00000164047	Immune response and inflammation

padj: Adjusted *p*-value using the Benjamini–Hochberg method to correct for multiple testing; Significance, Significance status with “S” indicating padj < 0.05 and “NS” meaning not significant, padj > 0.05.

## Data Availability

Processed data files (such as normalized count matrices or lists of DEGs), as well as other summary results, will be made available by the authors upon reasonable request.

## Supplementary Materials

The following supporting information can be downloaded at https://www.mdpi.com/article/10.3390/ijms26136152/s1.

## References

[1] Weidinger S., Beck L.A., Bieber T., Kabashima K., Irvine A.D. (2018). Atopic Dermatitis. Nat. Rev. Dis. Primers.

[2] Guttman-Yassky E., Renert-Yuval Y., Brunner P.M. (2025). Atopic Dermatitis. Lancet.

[3] Brunner P.M., Guttman-Yassky E., Leung D.Y.M. (2017). The Immunology of Atopic Dermatitis and Its Reversibility with Broad-Spectrum and Targeted Therapies. J. Allergy Clin. Immunol..

[4] Afshari M., Kolackova M., Rosecka M., Čelakovská J., Krejsek J. (2024). Unraveling the Skin; a Comprehensive Review of Atopic Dermatitis, Current Understanding, and Approaches. Front. Immunol..

[5] Pavel A.B., Zhou L., Diaz A., Ungar B., Dan J., He H., Estrada Y.D., Xu H., Fernandes M., Renert-Yuval Y. (2020). The Proteomic Skin Profile of Moderate-to-Severe Atopic Dermatitis Patients Shows an Inflammatory Signature. J. Am. Acad. Dermatol..

[6] Nakajima S., Nakamizo S., Nomura T., Ishida Y., Sawada Y., Kabashima K. (2024). Integrating Multi-Omics Approaches in Deciphering Atopic Dermatitis Pathogenesis and Future Therapeutic Directions. Allergy.

[7] Benedetto A.D., Rafaels N.M., McGirt L.Y., Ivanov A.I., Georas S.N., Cheadle C., Berger A.E., Zhang K., Vidyasagar S., Yoshida T. (2011). Tight Junction Defects in Patients with Atopic Dermatitis. J. Allergy Clin. Immunol..

[8] Otsuka A., Nomura T., Rerknimitr P., Seidel J.A., Honda T., Kabashima K. (2017). The Interplay between Genetic and Environmental Factors in the Pathogenesis of Atopic Dermatitis. Immunol. Rev..

[9] Möbus L., Rodriguez E., Harder I., Stölzl D., Boraczynski N., Gerdes S., Kleinheinz A., Abraham S., Heratizadeh A., Handrick C. (2021). Atopic Dermatitis Displays Stable and Dynamic Skin Transcriptome Signatures. J. Allergy Clin. Immunol..

[10] Bikle D.D. (2014). Vitamin D Metabolism, Mechanism of Action, and Clinical Applications. Chem. Biol..

[11] Schauber J., Gallo R.L. (2008). The Vitamin D Pathway: A New Target for Control of the Skin’s Immune Response?. Exp. Dermatol..

[12] Liu P.T., Stenger S., Li H., Wenzel L., Tan B.H., Krutzik S.R., Ochoa M.T., Schauber J., Wu K., Meinken C. (2006). Toll-like Receptor Triggering of a Vitamin D-Mediated Human Antimicrobial Response. Science.

[13] Grieco T., Paolino G., Moliterni E., Chello C., Sernicola A., Egan C.G., Morelli M., Nannipieri F., Battaglia S., Accoto M. (2024). Differential Expression of Proteins Involved in Skin Barrier Maintenance and Vitamin D Metabolism in Atopic Dermatitis: A Cross-Sectional, Exploratory Study. Int. J. Mol. Sci..

[14] Tsoi L.C., Rodriguez E., Degenhardt F., Baurecht H., Wehkamp U., Volks N., Szymczak S., Swindell W.R., Sarkar M.K., Raja K. (2019). Atopic Dermatitis Is an IL-13 Dominant Disease with Greater Molecular Heterogeneity Compared to Psoriasis. J. Investig. Dermatol..

[15] Sekita A., Kawasaki H., Fukushima-Nomura A., Yashiro K., Tanese K., Toshima S., Ashizaki K., Miyai T., Yazaki J., Kobayashi A. (2023). Multifaceted Analysis of Cross-Tissue Transcriptomes Reveals Phenotype–Endotype Associations in Atopic Dermatitis. Nat. Commun..

[16] Mitamura Y., Reiger M., Kim J., Xiao Y., Zhakparov D., Tan G., Rückert B., Rinaldi A.O., Baerenfaller K., Akdis M. (2023). Spatial Transcriptomics Combined with Single-Cell RNA-Sequencing Unravels the Complex Inflammatory Cell Network in Atopic Dermatitis. Allergy.

[17] Tsoi L.C., Rodriguez E., Stölzl D., Wehkamp U., Sun J., Gerdes S., Sarkar M.K., Hübenthal M., Zeng C., Uppala R. (2020). Progression of Acute-to-Chronic Atopic Dermatitis Is Associated with Quantitative Rather than Qualitative Changes in Cytokine Responses. J. Allergy Clin. Immunol..

[18] Grieco T., Moliterni E., Paolino G., Chello C., Sernicola A., Egan C.G., Nannipieri F., Battaglia S., Accoto M., Tirotta E. (2024). Association between Vitamin D Receptor Polymorphisms, Tight Junction Proteins and Clinical Features of Adult Patients with Atopic Dermatitis. Dermatol. Pract. Concept..

[19] Katsarou S., Makris M., Vakirlis E., Gregoriou S. (2023). The Role of Tight Junctions in Atopic Dermatitis: A Systematic Review. J. Clin. Med..

[20] Giannini S., Giusti A., Minisola S., Napoli N., Passeri G., Rossini M., Sinigaglia L. (2022). The Immunologic Profile of Vitamin D and Its Role in Different Immune-Mediated Diseases: An Expert Opinion. Nutrients.

[21] González-Tarancón R., Goñi-Ros N., Salvador-Rupérez E., Hernández-Martín Á., Izquierdo-Álvarez S., Puzo-Foncillas J., Gilaberte-Calzada Y. (2023). Association Between VDR and CYP24A1 Polymorphisms, Atopic Dermatitis, and Biochemical Lipid and Vitamin D Profiles in Spanish Population: Case-Control Study. JMIR Dermatol..

[22] Szabó L., Kapitány A., Somogyi O., Alhafez I., Gáspár K., Palatka R., Soltész L., Törőcsik D., Hendrik Z., Dajnoki Z. (2023). Antimicrobial Peptide Loss, Except for LL-37, Is Not Characteristic of Atopic Dermatitis. Acta Derm. Venereol..

[23] Plager D.A., Leontovich A.A., Henke S.A., Davis M.D.P., McEvoy M.T., Sciallis G.F., Pittelkow M.R. (2007). Early Cutaneous Gene Transcription Changes in Adult Atopic Dermatitis and Potential Clinical Implications. Exp. Dermatol..

[24] Knor T., Meholjić-Fetahović A., Mehmedagić A. (2011). Stratum Corneum Hydration and Skin Surface pH in Patients with Atopic Dermatitis. Acta Dermatovenerol. Croat..

[25] Suárez-Fariñas M., Tintle S.J., Shemer A., Chiricozzi A., Nograles K., Cardinale I., Duan S., Bowcock A.M., Krueger J.G., Guttman-Yassky E. (2011). Nonlesional Atopic Dermatitis Skin Is Characterized by Broad Terminal Differentiation Defects and Variable Immune Abnormalities. J. Allergy Clin. Immunol..

[26] Dessie E.Y., Ding L., Satish L., Mersha T.B. (2024). Co-Expression Network and Machine Learning Analysis of Transcriptomics Data Identifies Distinct Gene Signatures and Pathways in Lesional and Non-Lesional Atopic Dermatitis. J. Pers. Med..

[27] Wongvibulsin S., Sutaria N., Kannan S., Alphonse M.P., Belzberg M., Williams K.A., Brown I.D., Choi J., Roh Y.S., Pritchard T. (2021). Transcriptomic Analysis of Atopic Dermatitis in African Americans Is Characterized by Th2/Th17-Centered Cutaneous Immune Activation. Sci. Rep..

[28] Song J., Kim D., Lee S., Jung J., Joo J.W.J., Jang W. (2022). Integrative Transcriptome-Wide Analysis of Atopic Dermatitis for Drug Repositioning. Commun. Biol..

[29] Zhou J., Liang G., Liu L., Feng S., Zheng Z., Wu Y., Chen X., Li X., Wang L., Wang L. (2023). Single-Cell RNA-Seq Reveals Abnormal Differentiation of Keratinocytes and Increased Inflammatory Differentiated Keratinocytes in Atopic Dermatitis. J. Eur. Acad. Dermatol. Venereol..

[30] Makowska K., Nowaczyk J., Blicharz L., Waśkiel-Burnat A., Czuwara J., Olszewska M., Rudnicka L. (2023). Immunopathogenesis of Atopic Dermatitis: Focus on Interleukins as Disease Drivers and Therapeutic Targets for Novel Treatments. Int. J. Mol. Sci..

[31] Vu Y.H., Furue M., Tsuji G. (2021). The Role of Interleukin-24 in Atopic Dermatitis. Explor. Immunol..

[32] Zhu J., Wang Z., Chen F. (2019). Association of Key Genes and Pathways with Atopic Dermatitis by Bioinformatics Analysis. Med. Sci. Monit..

[33] Wang J., Huang Y., Wu X., Li D. (2024). MicroRNA-939 Amplifies Staphylococcus Aureus-Induced Matrix Metalloproteinase Expression in Atopic Dermatitis. Front. Immunol..

[34] Dębińska A. (2021). New Treatments for Atopic Dermatitis Targeting Skin Barrier Repair via the Regulation of FLG Expression. J. Clin. Med..

[35] Hatano Y., Elias P.M. (2023). “Outside-to-inside,” “inside-to-Outside,” and “Intrinsic” Endogenous Pathogenic Mechanisms in Atopic Dermatitis: Keratinocytes as the Key Functional Cells Involved in Both Permeability Barrier Dysfunction and Immunological Alterations. Front. Immunol..

[36] Torres T., Mendes-Bastos P., Cruz M.J., Duarte B., Filipe P., Lopes M.J.P., Gonçalo M. (2025). Interleukin-4 and Atopic Dermatitis: Why Does It Matter? A Narrative Review. Dermatol. Ther..

[37] Umar M., Sastry K.S., Al Ali F., Al-Khulaifi M., Wang E., Chouchane A.I. (2018). Vitamin D and the Pathophysiology of Inflammatory Skin Diseases. Skin Pharmacol. Physiol..

[38] Searing D.A., Leung D.Y. (2010). Vitamin D in Atopic Dermatitis, Asthma and Allergic Diseases. Immunol. Allergy Clin. N. Am..

[39] Pfisterer K., Wielscher M., Samardzic D., Weinzettl P., Symmank D., Shaw L.E., Campana R., Huang H.-J., Farlik M., Bangert C. (2023). Non-IgE-Reactive Allergen Peptides Deteriorate the Skin Barrier in House Dust Mite-Sensitized Atopic Dermatitis Patients. Front. Cell Dev. Biol..

[40] Lu R., Peng Z., Lian P., Wazir J., Gu C., Ma C., Wei L., Li L., Pu W., Liu J. (2023). Vitamin D Attenuates DNCB-Induced Atopic Dermatitis-like Skin Lesions by Inhibiting Immune Response and Restoring Skin Barrier Function. Int. Immunopharmacol..

[41] Margolis D.J. (2022). Atopic Dermatitis: Filaggrin and Skin Barrier Dysfunction. Br. J. Dermatol..

[42] van der Wal T., van Amerongen R. (2020). Walking the Tight Wire between Cell Adhesion and WNT Signalling: A Balancing Act for β-Catenin. Open Biol..

[43] Antonatos C., Mitsoudi D., Pontikas A., Akritidis A., Xiropotamos P., Georgakilas G.K., Georgiou S., Tsiogka A., Gregoriou S., Grafanaki K. (2025). Transcriptome-Wide Analyses Delineate the Genetic Architecture of Expression Variation in Atopic Dermatitis. HGG Adv..

[44] Vogel C., Marcotte E.M. (2012). Insights into the Regulation of Protein Abundance from Proteomic and Transcriptomic Analyses. Nat. Rev. Genet..

[45] Wilhelm M., Schlegl J., Hahne H., Gholami A.M., Lieberenz M., Savitski M.M., Ziegler E., Butzmann L., Gessulat S., Marx H. (2014). Mass-Spectrometry-Based Draft of the Human Proteome. Nature.

[46] Perl K., Ushakov K., Pozniak Y., Yizhar-Barnea O., Bhonker Y., Shivatzki S., Geiger T., Avraham K.B., Shamir R. (2017). Reduced Changes in Protein Compared to mRNA Levels across Non-Proliferating Tissues. BMC Genom..

[47] Guillemin A., Kumar A., Wencker M., Ricci E.P. (2022). Shaping the Innate Immune Response Through Post-Transcriptional Regulation of Gene Expression Mediated by RNA-Binding Proteins. Front. Immunol..

[48] Goleva E., Calatroni A., LeBeau P., Berdyshev E., Taylor P., Kreimer S., Cole R.N., Leung D.Y.M. (2020). Skin Tape Proteomics Identifies Pathways Associated with Transepidermal Water Loss and Allergen Polysensitization in Atopic Dermatitis. J. Allergy Clin. Immunol..

[49] Cole C., Kroboth K., Schurch N.J., Sandilands A., Sherstnev A., O’Regan G.M., Watson R.M., McLean W.H.I., Barton G.J., Irvine A.D. (2014). Filaggrin-Stratified Transcriptomic Analysis of Pediatric Skin Identifies Mechanistic Pathways in Patients with Atopic Dermatitis. J. Allergy Clin. Immunol..

[50] Rusiñol L., Puig L. (2024). Multi-Omics Approach to Improved Diagnosis and Treatment of Atopic Dermatitis and Psoriasis. Int. J. Mol. Sci..

[51] Bratu D., Boda D., Caruntu C. (2023). Genomic, Epigenomic, Transcriptomic, Proteomic and Metabolomic Approaches in Atopic Dermatitis. Curr. Issues Mol. Biol..

[52] Furue M., Chiba T., Tsuji G., Ulzii D., Kido-Nakahara M., Nakahara T., Kadono T. (2017). Atopic Dermatitis: Immune Deviation, Barrier Dysfunction, IgE Autoreactivity and New Therapies. Allergol. Int..

[53] Elias P.M., Hatano Y., Williams M.L. (2008). Basis for the Barrier Abnormality in Atopic Dermatitis: Outside-inside-Outside Pathogenic Mechanisms. J. Allergy Clin. Immunol..

[54] Hanifin J.M., Thurston M., Omoto M., Cherill R., Tofte S.J., Graeber M. (2001). The Eczema Area and Severity Index (EASI): Assessment of Reliability in Atopic Dermatitis. EASI Evaluator Group. Exp. Dermatol..

