# Supplementary material for: Transcriptomic Profiling of Lesional and Perilesional Skin in Atopic Dermatitis Suggests Barrier Dysfunction, Inflammatory Activation, and Alterations to Vitamin D Metabolism
Source: Int J Mol Sci. 2025 Jun 26;26(13):6152. doi: 10.3390/ijms26136152 (PMC12249519; doi:10.3390/ijms26136152)

Supplementary Figure S1. Volcano plot of differentially expressed genes (DEGs) between intralesional (IL) and perilesional (PL) skin in atopic dermatitis. Each dot represents a gene plotted according to its $\log_2$ fold change (x-axis) and statistical significance ($-\log_{10}$ *p*-value, y-axis). Red dots indicate DEGs with adjusted *p*-value < 0.05 and $|\log_2$ fold change$| > 1$ (significant). Blue dots represent genes with adjusted *p*-value < 0.05 but $|\log_2$ fold change$| \leq 1$. Green dots indicate genes with $|\log_2$ fold change$| > 1$ but not statistically significant (adjusted *p*-value ≥ 0.05). Grey dots represent genes with no significant differential expression. Notable upregulated and downregulated genes are labeled. In total, 42,433 genes were analyzed.

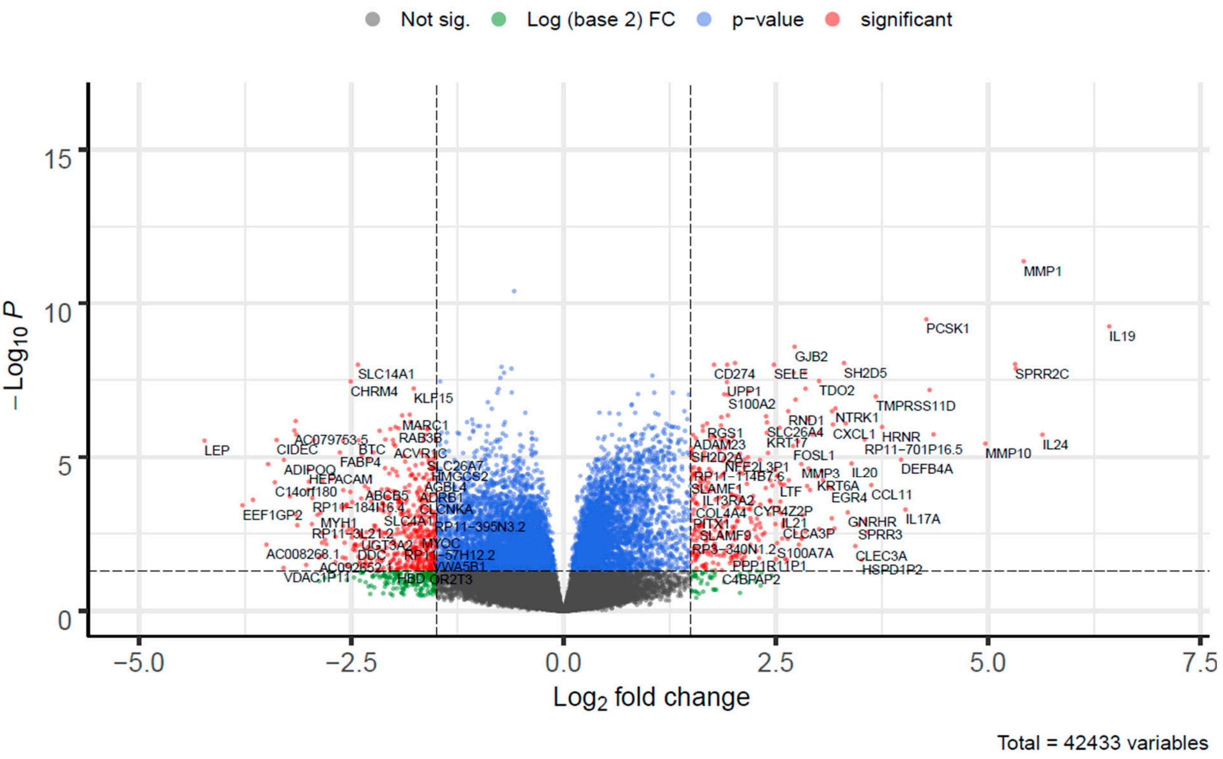

Supplement: Supplementary file 1 [file ijms-26-06152-s001.zip › Supplementary Figure S1a.pdf]
