# Supplementary material for: Transcriptomic Profiling of Lesional and Perilesional Skin in Atopic Dermatitis Suggests Barrier Dysfunction, Inflammatory Activation, and Alterations to Vitamin D Metabolism
Source: Int J Mol Sci. 2025 Jun 26;26(13):6152. doi: 10.3390/ijms26136152 (PMC12249519; doi:10.3390/ijms26136152)

Supplementary Figure S2. Expression levels of Atopic Dermatitis-related genes in Intralesional (IL) and Perilesional (PL) Skin Biopsies. Boxplots displaying the distribution of normalized read counts for 12 genes (IL, yellow boxes and PL, blue boxes): (a) *CDH1*, (b) *TJP1*, (c) *CTNNA1*, (d) *CTNNB1*, (e) *CLDN1*, (f) *CGNL1*, (g) *FLG2*, (h) *OCLN*, (i) *CYP27B1*, (j) *CYP24A1*, (k) *VDR*, and (l) *CAMP*. The boxplots illustrate the median (center line), interquartile range (box), and range of the data (whiskers). Individual data points are represented as red dots, with outliers shown as open circles.

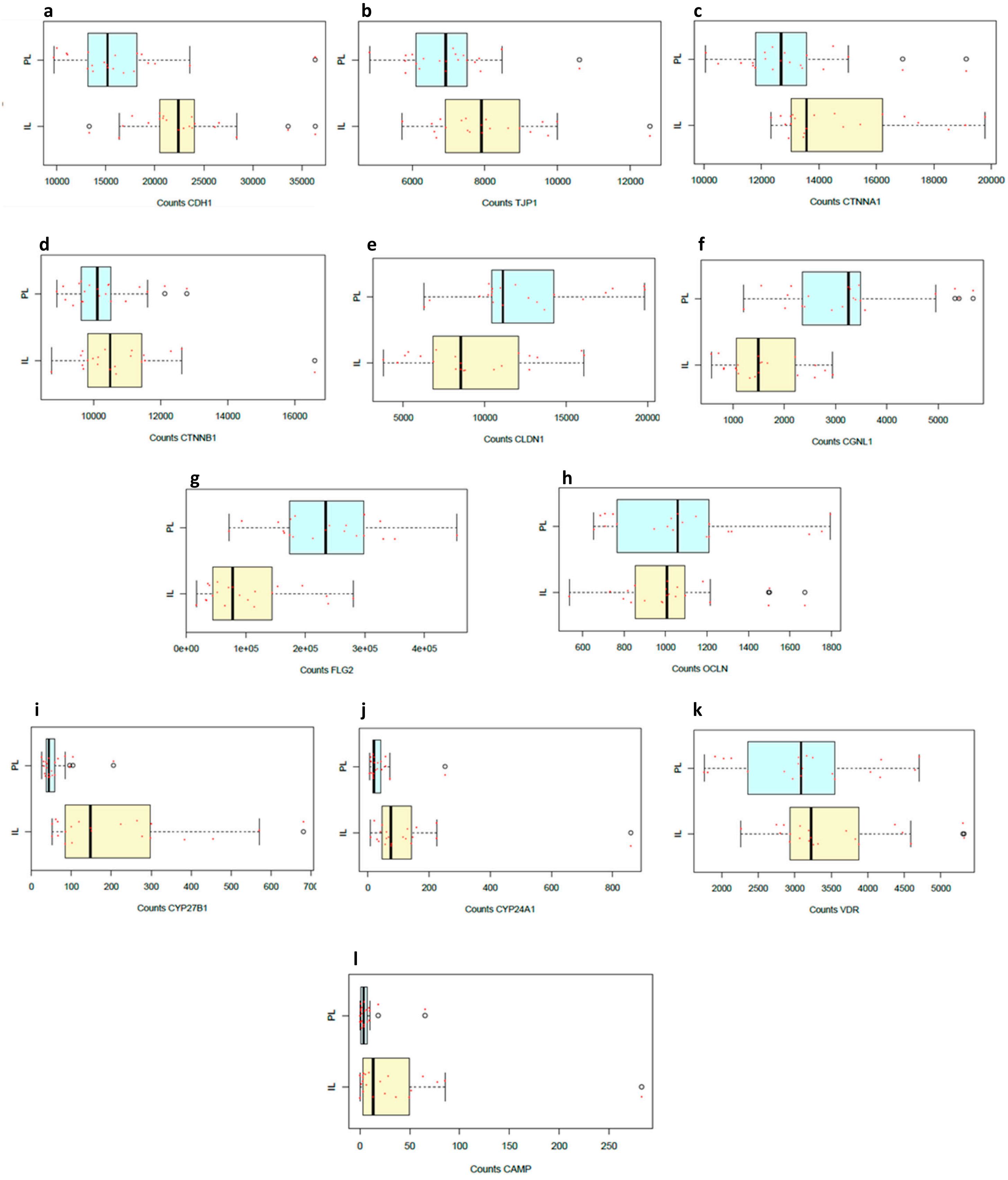

Supplement: Supplementary file 1 [file ijms-26-06152-s001.zip › Supplementary Figure S2.pdf]
