# Supplementary material for: Transcriptomic Profiling of Lesional and Perilesional Skin in Atopic Dermatitis Suggests Barrier Dysfunction, Inflammatory Activation, and Alterations to Vitamin D Metabolism
Source: Int J Mol Sci. 2025 Jun 26;26(13):6152. doi: 10.3390/ijms26136152 (PMC12249519; doi:10.3390/ijms26136152)

Supplementary Figure S4. Significant gene expression correlation in Perilesional samples (A) and in Intralesional samples (B). Heatmaps show significant Pearson correlation coefficients (r) between genes. Only significant correlations are shown ($p < 0.05$). Color intensity reflects the strength and direction of correlation: red indicates positive and blue negative correlations. White squares indicate non-significant values.

A

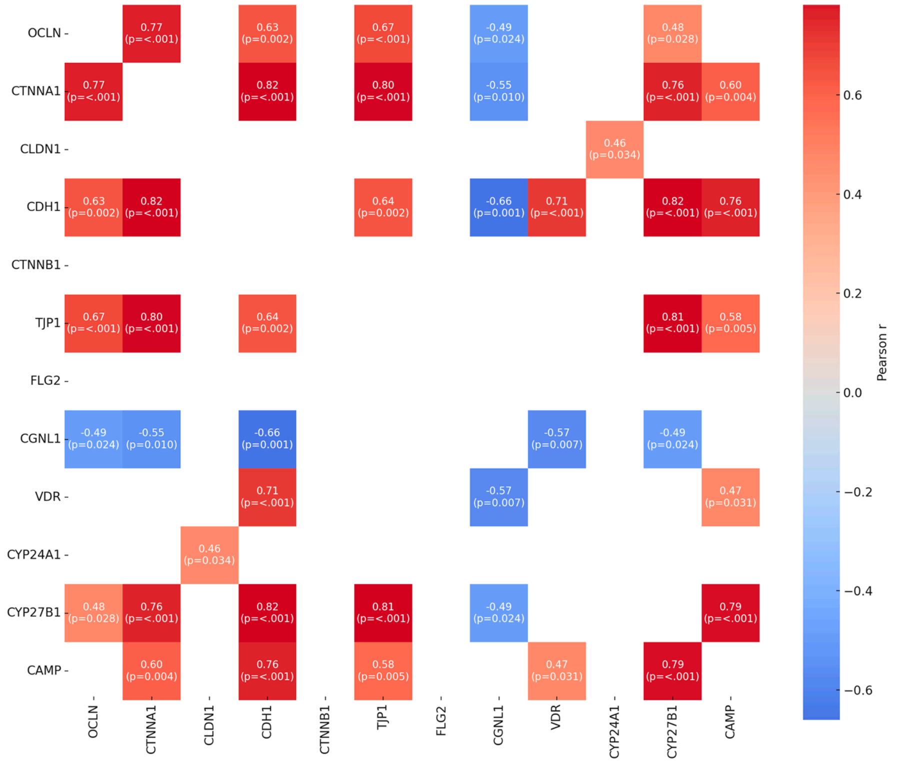

B

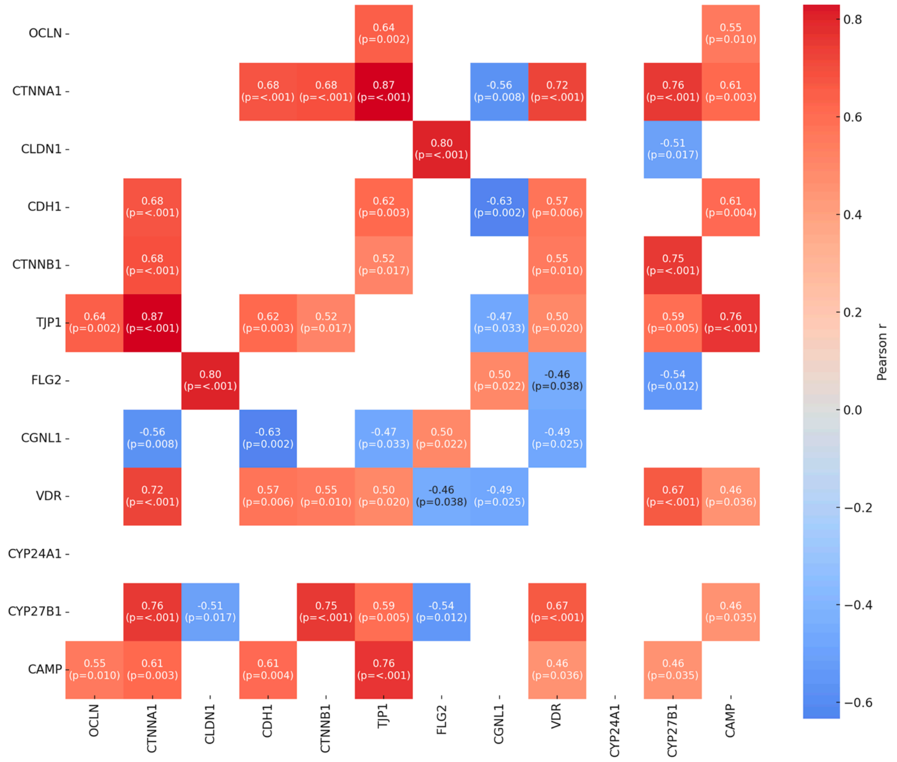

Supplement: Supplementary file 1 [file ijms-26-06152-s001.zip › Supplementary Figure S3.pdf]
