# Supplementary material for: Transcriptomic Profiling of Lesional and Perilesional Skin in Atopic Dermatitis Suggests Barrier Dysfunction, Inflammatory Activation, and Alterations to Vitamin D Metabolism
Source: Int J Mol Sci. 2025 Jun 26;26(13):6152. doi: 10.3390/ijms26136152 (PMC12249519; doi:10.3390/ijms26136152)
Supplement: Supplementary file 1 [file Supplementary Figure S1b.pdf › Supplementary Figure S1b.pdf]

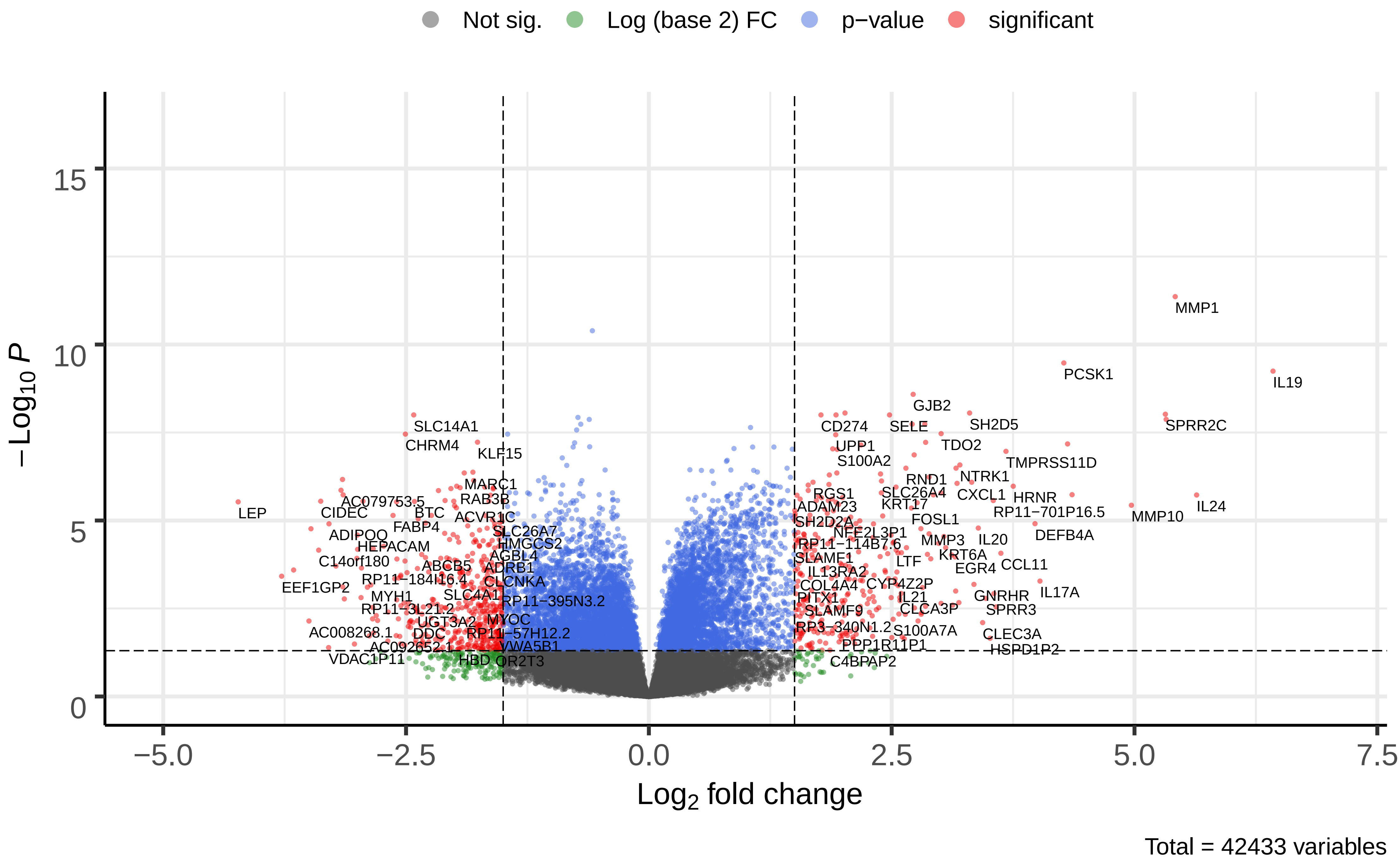

Not sig.
Log (base 2) FC
p−value
significant
−Log10 P
Log2 fold change
Total = 42433 variables
0
5
10
15
−5.0
−2.5
0.0
2.5
5.0
7.5
MMP1
PCSK1
IL19
GJB2
SPRR2C
SH2D5
CD274
SELE
TDO2
UPP1
S100A2
TMPRSS11D
RND1
NTRK1
RGS1
SLC26A4
CXCL1
HRNR
KRT17
ADAM23
RP11−701P16.5
MMP10
IL24
FOSL1
SH2D2A
NFE2L3P1
DEFB4A
RP11−114B7.6
MMP3
IL20
KRT6A
SLAMF1
LTF
EGR4
CCL11
IL13RA2
COL4A4
CYP4Z2P
IL17A
PITX1
IL21
GNRHR
SLAMF9
CLCA3P
SPRR3
RP3−340N1.2
S100A7A
CLEC3A
PPP1R11P1
HSPD1P2
C4BPAP2
SLC14A1
CHRM4
KLF15
MARC1
RAB3B
AC079753.5
LEP
CIDEC
BTC
ACVR1C
FABP4
ADIPOQ
SLC26A7
HEPACAM
HMGCS2
AGBL4
C14orf180
ABCB5
ADRB1
RP11−184I16.4
CLCNKA
EEF1GP2
MYH1
SLC4A1
RP11−395N3.2
RP11−3L21.2
UGT3A2
MYOC
AC008268.1
DDC
RP11−57H12.2
AC092652.1
VWA5B1
VDAC1P11
HBD
OR2T3
